# Predictive Value of the Fibrinogen to Gamma-Glutamine Transferase Ratio in the Long-Term Outcome in Patients with Coronary Heart Disease: A Retrospective Cohort Study

**DOI:** 10.31083/j.rcm2412369

**Published:** 2023-12-26

**Authors:** Ju Yan, Chang-Jiang Deng, Xuan Min, Yi Ning, Ming-Yuan Wang, Si-Fan Wang, Mikereyi Aimaitijiang, Ying-Ying Zheng, Xiang Xie, Yi-Tong Ma

**Affiliations:** ^1^Department of Cardiology, The First Affiliated Hospital of Xinjiang Medical University, 830054 Urumqi, Xinjiang, China

**Keywords:** mortality, coronary heart disease (CHD), fibrinogen/γ-glutamine transferase (FGR), fibrinogen, γ-glutamine transferase, long-term outcome

## Abstract

**Background:**

The ratio of fibrinogen to γ-glutamine transferase (FGR) was used to predict long-term prognosis in patients with coronary heart disease (CHD).

**Methods:**

A total of 5638 patients with CHD who were hospitalized from January 2008 to December 2016 were retrospectively enrolled in the study. The mean follow-up time was 35.9 ± 22.5 months. The follow-up endpoints were major cardiac and cerebrovascular adverse events (MACCE). The optimal FGR cut-off value was determined and divided into high- and low-FGR groups according to the receiver operating characteristic (ROC) curve. Statistical methods were used to compare the differences between the two groups and their prognoses to determine whether FGR can predict prognosis in patients with CHD. The traditional predictors were incorporated into the logistic regression model to observe the correlation between these indicators and all-cause mortality (ACM) events. We compared the prediction performance of FGR and traditional predictors on the occurrence of ACM events by ROC curves.

**Results:**

The optimal cut-off value was determined via a ROC analysis (FGR = 1.22, *p* = 0.002), and subjects were classified into high and low FGR groups. The follow-up found that the incidence of MACCE in the high FGR group was higher than that in the low FGR group. The COX multivariate regression model showed that high FGR was independently correlated with the occurrence of MACCE. In addition, the Kaplan–Meier survival curve showed that the risk of events was significantly increased in the group with high FGR. With increases in the FGR ratio, the risk of MACCE was increased. The ROC curve revealed that the risk of ACM was statistically different between the FGR and the traditional risk factor model (*p* = 0.002), (Fibrinogen (*p* = 0.008), γ-glutamine transferase (GGT) (*p* = 0.004), and N-terminal pro brain natriuretic peptide (NT-ProBNP) (*p* = 0.024)). The comparison between other different models were not statistically significant (*p* > 0.05). The area under the FGR model curve was larger than that of the traditional risk factors, fibrinogen, GGT and NT-ProBNP models.

**Conclusions:**

High FGR can increase the risk of MACCE in patients with CHD; additionally, it can be used as a new biomarker for long-term prognosis in CHD patients.

**Clinical Trial Registration:**

All details of this study are registered on the website (http://www.chictr.org.cn), registration number: ChiCTR-ORC-16010153.

## 1. Introduction

In the 1940s, cardiovascular disease was the leading cause of death in the United States, accounting for half of all deaths. Approximately every 40 seconds, someone in the United States has an acute heart attack. Throughout Europe, coronary heart disease is responsible for approximately 1.8 million deaths per year [1]. According to the 2017 Global Burden of Disease study, ischemic heart disease is one of the most disabling diseases. Circulatory diseases caused 1779 million deaths worldwide in 2017 (an increase of 44.9% compared to 1990) [2]. Joseph P *et al*. [3] showed that while age-standardized mortality from cardiovascular disease has declined, the absolute number of deaths continues to increase, with most deaths now occurring in low- and middle-income countries. According to the China Cardiovascular Health and Disease Report 2020, cardiovascular disease is the leading cause of death in urban and rural areas in China, accounting for 46.66% in rural areas and 43.81% in urban areas. The economic burden of cardiovascular disease on residents and society is increasing [4]. With the continuous development of society, the diagnosis and treatment of coronary heart disease are also continually improving. For example, the development of body surface imaging (cardiac magnetic resonance imaging (MRI), myocardial metabolic imaging and Single-Photon Emission Computed Tomography (SPECT)) and endovascular imaging (optical coherence tomography (OCT), intravascular ultrasound (IVUS) and fractional flow reserve (FFR)); the gold standard of coronary angiography in the diagnosis of coronary heart disease (CHD), the establishment and use of stem cells and degradable stents in the treatment of CHD; and large sample clinical and genetic databases have all resulted in improved outcomes for patients with CHD. However, the numbers of CHD patients and deaths in low- and middle-income countries are still on the rise, and the burden of disease on society and CHD patients is still significant.

Clinical studies have confirmed that traditional risk factors for CHD are associated with poor prognosis of CHD, such as age, sex, smoking, drinking, history of diabetes mellitus, history of hypertension, and risk factors such as lipid levels. With the advent of cardiac markers, N-terminal pro brain natriuretic peptide (NT-proBNP) and troponin were also associated with poor prognoses of CHD. Additionally, Fibrinogen and γ-glutamine transferase (GGT) are also associated with poor prognosis but the predictive value of their ratio (fibrinogen/γ-glutamine transferase, FGR) for the prognosis of patients with CHD is unknown. Therefore, this study used FGR to predict the poor prognosis of CHD patients through the use of long-term follow-up to determine whether FGR can predict the occurrence of major cardiac and cerebrovascular adverse events (MACCE), major adverse cardiovascular events (MACE), all-cause mortality (ACM) and cardiogenic mortality (CM) in CHD patients, by comparing our novel cardiac marker FGR with traditional risk factors, such as NT-proBNP, to further improve clinical diagnosis and treatment.

## 2. Methods

### 2.1 Inclusion and Exclusion Criteria

**Fig. 1.**
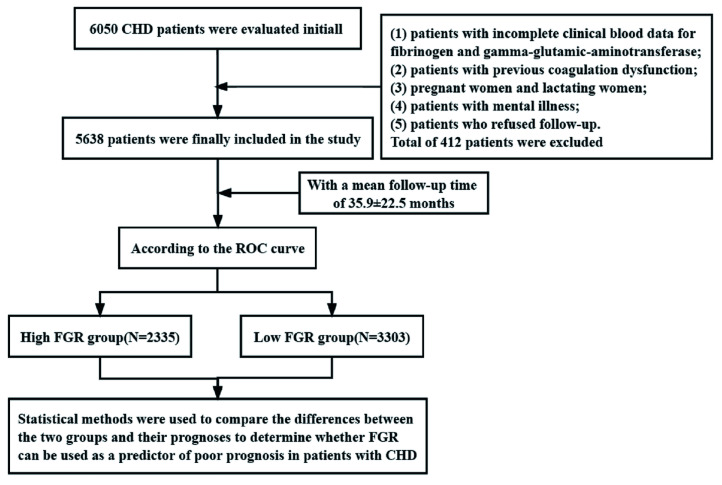
**Inclusion of research objects and flow chart**. CHD, coronary heart disease; ROC, receiver operating characteristic; FGR, fibrinogen/γ-glutamine transerase.

A total of 6050 patients who were hospitalized for CHD at the Heart Center of the First Affiliated Hospital of Xinjiang Medical University from Jan 1, 2008 to Dec 31, 2016 were enrolled in the study. The following inclusion criteria were used: patients who were diagnosed with coronary heart disease after completing coronary angiography after admission, including stable angina, unstable angina, acute ST-segment elevation myocardial infarction and nonacute ST-segment elevation myocardial infarction. The following exclusion criteria were used: (1) patients with incomplete clinical blood data for fibrinogen and gamma-glutamic-aminotransferase; (2) patients with previous coagulation dysfunction; (3) pregnant women and lactating women; (4) patients with mental illness; and (5) patients who refused follow-up. After passing the inclusion as well as exclusion criteria, 5638 patients with CHD were finally enrolled in the study (specific process is described in Fig. 1).

### 2.2 Research Methods

General and clinical data of patients were collected from the electronic medical record system of the hospital. General information included age, sex, previous smoking and alcohol use, past history of hypertension and diabetes mellitus (DM). Clinical data included fibrinogen, blood urea nitrogen (BUN), γ-glutamine transferase, serum creatinine (Scr), uric acid (UA), total cholesterol (TC), high-density lipoprotein cholesterol (HDL-C), triglycerides (TG), low-density lipoprotein cholesterol (LDL-C), left ventricular ejection fraction (LVEF) and coronary artery and stent factors (lesion blood vessel number, pre-dilation, post-dilation, dilation pressure, the choice of using a novel coronary stent, chronic coronary total occlusions (CTO), multivessel lesions, stent length, stent diameter and the number of stents) and drug use (calcium antagonists, beta-blockers, angiotensin II receptor antagonists and statins). The diagnostic criteria for hypertension included systolic blood pressure ≥140 mmHg, diastolic blood pressure ≥90 mmHg, or both conditions occurring simultaneously, as well as the use of blood pressure-lowering drugs in the last two weeks [5]. The following criteria were used for a diagnosis of DM: fasting blood glucose ≥7.0 mmol/L, random blood glucose or two hours postprandial blood glucose ≥11.1 mmol/L, or hBA1c ≥6.5%, or recent use of hypoglycemic drugs or insulin [6].

### 2.3 Follow-Up

The mean follow-up was (35.9 ± 22.5) months by telephone, outpatient visits, and re-hospitalizations. The primary endpoint event at follow-up was a composite of MACCE and major adverse cardiovascular events (MACE), MACCE: including MACE and stroke, MACE including nonfatal myocardial infarction, angina attack with revascularization, and cardiogenic mortality (CM). The secondary endpoint event was all-cause mortality (ACM).

### 2.4 Statistical Methods

SPSS 21.0 statistical analysis software (IBM Corp., Armonk, NY, USA) was used for data analysis and processing. The number of cases (as percentages) was used in counting the data. First, the measured data were tested for a normal distribution. The measured data that matched or nearly matched the normal distribution were used $\bar{x}$ *± s*, and the non-normal measurement data were used for median and quad spacing (M, P25 to P75). The independent sample *T*-test was used to compare the measurement data of FGR between the two groups, and the χ^2^ test was used to compare the count data. Multivariate COX models were used to determine the independent parameters of long-term mortality and MACCE. The Kaplan–Meier method was used to construct the cumulative survival curves of the endpoints, and the log-rank test was used for the comparisons. The correlation with ACM was observed by logistic regression models incorporating traditional risk factors as well as relevant indicators. The predictive efficacy of different cardiac biomarkers was compared by receiver operating characteristic (ROC) curves. A value of *p* < 0.05 was considered to be statistically significant.

## 3. Results

From the receiver operating curve, the optimal cut-off value (FGR = 1.22, *p* = 0.002) was determined, and the patients were classified into high and low FGR groups according to the optimal cut-off value. Differences were found between the two groups in age, sex, history of hypertension, triglyceride levels, total cholesterol levels, high-density lipoprotein levels, low-density lipoprotein levels, left ventricular ejection fraction, number of vessels with coronary artery disease, poststent dilation, poststent dilation pressure and the amount of multivessel coronary disease. The high FGR group was older than the low FGR group; additionally, the high FGR group had more females, had more hypertension, lower triglyceride levels, lower total cholesterol levels, higher HDL-C levels, lower LDL-C levels, higher left ventricular ejection fraction, more coronary lesions, less need for post-dilation, less need for post-stenting, lower coronary dilatation pressure and a higher number of multivessel coronary lesions, according to Tables 1,2.

**Table 1.** **The comparison of measurement data between the two groups of clinical and laboratory**.

	Low FGR group	High FGR group	*p*	T	95% CI	
	(N = 3303)	(N = 2335)			Lower	Upper
Age (years)	59.127 ± 10.868	59.981 ± 10.699	0.003	–2.924	–1.426	–0.281
BUN (mmol/L)	5.543 ± 1.684	5.492 ± 1.699	0.262	1.123	–0.038	0.141
Scr (mmol/L)	76.043 ± 20.401	75.779 ± 20.584	0.634	0.477	–0.823	1.351
UA (mmol/L)	324.252 ± 89.630	322.598 ± 90.043	0.496	0.680	–3.112	6.420
TG (mmol/L)	2.062 ± 1.362	1.673 ± 1.067	<0.001	11.875	0.325	0.454
TC (mmol/L)	3.991 ± 1.125	3.924 ± 1.086	0.028	2.204	0.007	0.127
HDL-C (mmol/L)	1.005 ± 0.504	1.045 ± 0.463	0.003	–2.949	–0.066	–0.013
LDL-C (mmol/L)	2.490 ± 0.902	2.425 ± 0.929	0.009	2.611	0.016	0.114
LVEF (%)	60.897 ± 7.230	61.390 ± 6.746	0.013	–2.485	–0.882	–0.104
Number of lesion vessels (n)	1.980 ± 0.847	2.050 ± 0.844	0.002	–3.034	–0.114	–0.025
Pre-dilation pressure (atm)	11.818 ± 2.428	11.841 ± 2.662	0.820	–0.228	–0.216	0.171
Post-dilation pressure (atm)	13.980 ± 3.593	13.712 ± 3.461	0.018	2.377	0.047	0.490
Stent length (mm)	27.953 ± 6.935	27.974 ± 7.041	0.910	–0.113	–0.391	0.349
Stent diameter (mm)	2.846 ± 0.377	2.852 ± 0.369	0.547	–0.602	–0.026	0.014
Number of stents (n)	1.038 ± 0.207	1.049 ± 0.248	0.076	–1.775	–0.023	0.001

*p* < 0.05 was statistically significant. FGR, fibrinogen to γ-glutamine transferase; BUN, blood urea nitrogen; Scr, serum creatinine; UA, uric acid; TG, triglycerides; TC, total cholesterol; HDL-C, high-density lipoprotein cholesterol; LDL-C, low-density lipoprotein cholesterol; LVEF, left ventricular ejection fraction.

**Table 2.** **The comparison of enumeration data between the two groups of clinical and laboratory**.

			FGR		χ ^2^	*p*-value
			Low group (N = 3303)	High group (N = 2335)		
Sex [n (%)]		men	2512 (76.1%)	1681 (72%)	11.834	0.001
		women	791 (23.9%)	654 (28%)		
DM [n (%)]		0	2487 (75.3%)	1780 (76.2%)	0.651	0.420
		1	816 (24.7%)	555 (23.8%)		
Hypertension [n (%)]		0	1946 (58.9%)	1298 (55.6%)	6.199	0.013
		1	1357 (41.1%)	1037 (44.4%)		
Use of drugs						
	Calcium antagonist [n (%)]	0	2910 (88.6%)	2055 (88.6%)	0.001	0.981
		1	376 (11.4%)	265 (11.4%)		
	β-blocker [n (%)]	0	1949 (59.3%)	1396 (60.1%)	0.379	0.538
		1	1339 (40.7%)	927 (39.9%)		
	Angiotensin II receptor blockers [n (%)]	0	2518 (76.7%)	1810 (78%)	1.278	0.258
		1	765 (23.3%)	511 (22%)		
	Statins [n (%)]	0	1463 (44.7%)	1081 (46.7%)	2.298	0.130
		1	1811 (55.3%)	1232 (53.3%)		
Coronary artery and stent status						
	Pre-dilation [n (%)]	0	436 (13.2%)	329 (14.1%)	0.936	0.333
		1	2867 (86.8%)	2005 (85.9%)		
	Post-dilation [n (%)]	0	1201 (36.4%)	921 (39.5%)	5.596	0.018
		1	2102 (63.6%)	1413 (60.5%)		
	A novel coronary stent [n (%)]	0	199 (6%)	127 (5.4%)	0.855	0.355
		1	2104 (94%)	2207 (94.6%)		
	CTO [n (%)]	0	2559 (77.5%)	1763 (75.5%)	2.876	0.090
		1	744 (22.5%)	571 (24.5%)		
	Multivessel coronary disease [n (%)]	0	1218 (36.9%)	776 (33.2%)	7.874	0.005
		1	2085 (63.1%)	1558 (66.8%)		

*p* < 0.05 was statistically significant. DM, diabetes mellitus; CTO, chronic coronary total occlusions; FGR, fibrinogen/γ-glutamine transferase.

Following a mean follow-up time of 35.9 ± 22.5 months, as shown in Table 3, there were 291 total all-cause deaths, 234 total cardiac deaths, 799 total MACCE and 729 total MACE at long-term follow-up. There was an increased incidence of MACCE (15.8% *vs.* 13%, *p* = 0.004), MACE (14.6% *vs.* 11.7%, *p* = 0.001), ACM (6.3% *vs.* 4.3%, *p* = 0.001) and CM (5.3% *vs.* 3.4%, *p* < 0.001) in the high FGR group compared to the low FGR group, respectively.

**Table 3.** **Clinical outcomes comparison between Low and High FGR groups**.

		FGR		χ ^2^	*p*-value
		Low group (N = 3303)	High group (N = 2335)		
MACCEs	0	2872 (87%)	1967 (84.2%)	8.268	0.004
	1	431 (13%)	368 (15.8%)		
MACEs	0	2916 (88.3%)	1993 (85.4%)	10.432	0.001
	1	387 (11.7%)	342 (14.6%)		
ACM	0	3160 (95.7%)	2187 (93.7%)	11.278	0.001
	1	143 (4.3%)	148 (6.3%)		
CM	0	3192 (96.6%)	2212 (94.7%)	12.506	<0.001
	1	111 (3.4%)	123 (5.3%)		

*p* < 0.05 was statistically significant. MACCEs, major cardiac and cerebrovascular adverse events; MACEs, major adverse cardiovascular events; ACM, all-cause mortality; CM, cardiogenic mortality; FGR, fibrinogen/γ-glutamine transferase.

**Fig. 2.**
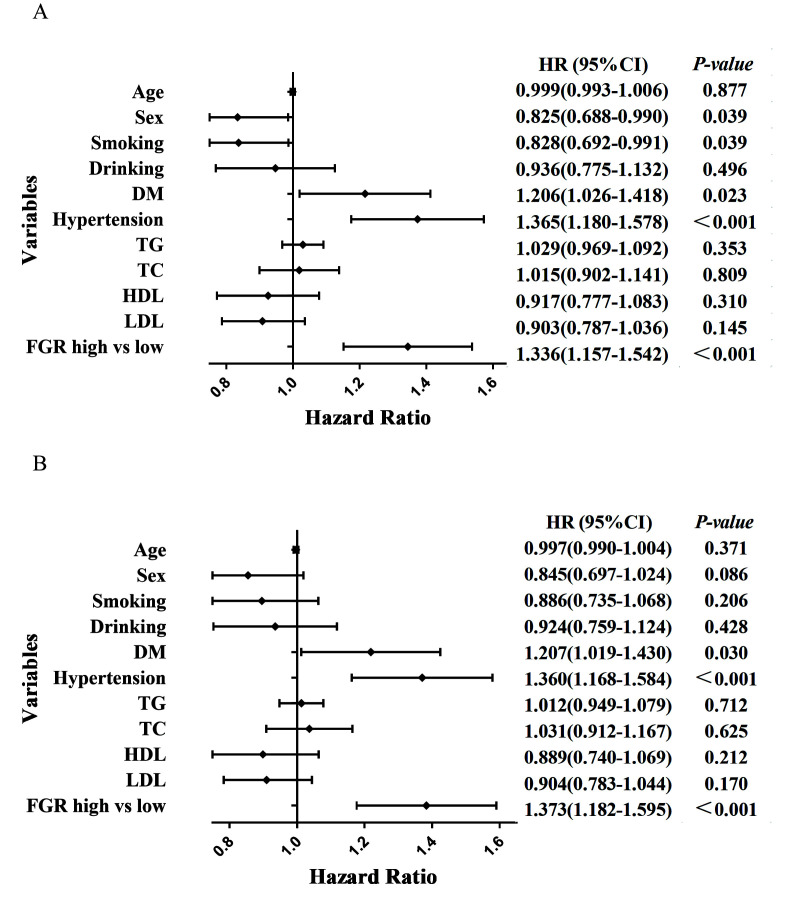
**Forest plots of MACCE and MACE patients based on COX regression model results**. (A) Forest plots of MACCE patients based on COX regression model results. (B) Forest plots of MACE patients based on COX regression model results. MACCE, major cardiac and cerebrovascular adverse events; MACE, major adverse cardiovascular events; DM, diabetes mellitus; TG, triglycerides; TC, total cholesterol; HDL, high-density lipoprotein; LDL, low-density lipoprotein; FGR, fibrinogen/γ-glutamine transferase; HR, hazard ratio.

**Fig. 3.**
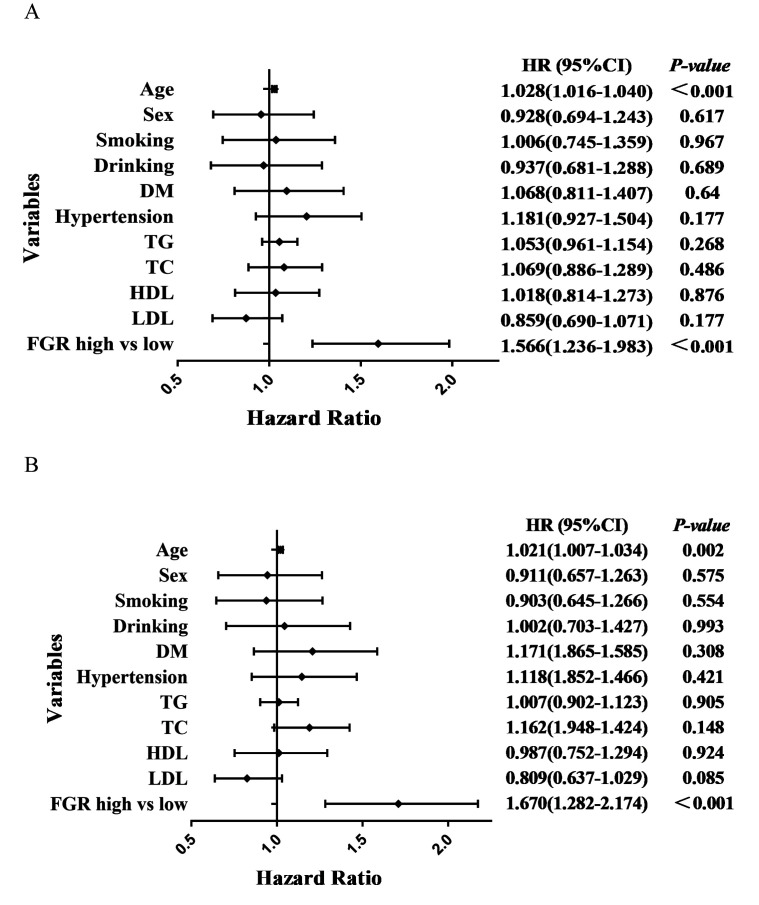
**Forest plots of ACM and CM patients based on COX regression model results**. (A) Forest plots of ACM patients based on COX regression model results. (B) Forest plots of CM patients based on COX regression model results. ACM, all-cause mortality; CM, cardiogenic mortality; DM, diabetes mellitus; TG, triglycerides; TC, total cholesterol; HDL, high-density lipoprotein; LDL, low-density lipoprotein; FGR, fibrinogen/γ-glutamine transferase; HR, hazard ratio.

We demonstrated the correlation between the two groups of FGR and MACCE, MACE, ACM and CM via the COX multifactorial regression model after correcting for age, sex, history of smoking, history of alcohol consumption, previous diabetes and hypertension, triglycerides, total cholesterol, high- and low-density lipoproteins and low-density lipoproteins. High FGR, previous history of hypertension and a history of diabetes were independently associated with the occurrence of MACCE, with high FGR being 1.34 times more likely than low FGR to experience MACCE (hazard ratio (HR) = 1.336 [1.157–1.542], *p* < 0.001) (Table 4 and Fig. 2A). High FGR and a previous history of hypertension and diabetes were independently associated with the occurrence of MACE, with high FGR being 1.37 times more likely than low FGR to develop MACE (HR = 1.373 [1.182–1.595], *p* < 0.001) (Table 4 and Fig. 2B). High FGR, age and ACM occurrence were independently associated, and high FGR was 1.57 times more likely than low FGR to occur with ACM (HR = 1.566 [1.236–1.983], *p* < 0.001) (Table 5 and Fig. 3A). Additionally, high FGR, age and CM occurrence were independently associated, and high FGR was 1.67 times more likely to cause CM than low FGR (HR = 1.670 [1.282–2.174], *p* < 0.001) (Table 5 and Fig. 3B).

**Table 4.** **COX regression analysis results for CHD MACCEs and MACEs**.

	MACCEs					MACEs				
	B	SE	χ ^2^	*p*-value	HR (95% CI)	B	SE	χ ^2^	*p*-value	HR (95% CI)
Age	–0.001	0.004	0.024	0.877	0.999 (0.993–1.006)	–0.003	0.004	0.799	0.371	0.997 (0.990–1.004)
Sex	–0.192	0.093	4.270	0.039	0.825 (0.688–0.990)	–0.169	0.098	2.952	0.086	0.845 (0.697–1.024)
Smoking	–0.189	0.092	4.257	0.039	0.828 (0.692–0.991)	–0.121	0.095	1.600	0.206	0.886 (0.735–1.068)
Drinking	–0.066	0.097	0.464	0.496	0.936 (0.775–1.132)	–0.079	0.100	0.627	0.428	0.924 (0.759–1.124)
DM	0.188	0.083	5.160	0.023	1.206 (1.026–1.418)	0.188	0.086	4.736	0.030	1.207 (1.019–1.430)
Hypertension	0.311	0.074	17.503	<0.001	1.365 (1.180–1.578)	0.308	0.078	15.691	<0.001	1.360 (1.168–1.584)
TG	0.028	0.031	0.861	0.353	1.029 (0.969–1.092)	0.012	0.033	0.136	0.712	1.012 (0.949–1.079)
TC	0.014	0.060	0.058	0.809	1.015 (0.902–1.141)	0.031	0.063	0.239	0.625	1.031 (0.912–1.167)
HDL-C	–0.086	0.085	1.032	0.310	0.917 (0.777–1.083)	–0.117	0.094	1.557	0.212	0.889 (0.740–1.069)
LDL-C	–0.102	0.070	2.127	0.145	0.903 (0.787–1.036)	–0.101	0.073	1.883	0.170	0.904 (0.783–1.044)
FGR high *vs.* low	0.289	0.073	15.674	<0.001	1.336 (1.157–1.542)	0.317	0.076	17.217	<0.001	1.373 (1.182–1.595)

*p* < 0.05 was statistically significant. HR, hazard ratio; B, regression coefficient; SE, standard error; MACCEs, major cardiac and cerebrovascular adverse events; MACEs, major adverse cardiovascular events; CHD, coronary heart disease; DM, diabetes mellitus; TG, triglycerides; TC, total cholesterol; HDL-C, high-density lipoprotein cholesterol; LDL-C, low-density lipoprotein cholesterol; FGR, fibrinogen/γ-glutamine transferase.

**Table 5.** **COX regression analysis results for CHD ACM and CM**.

	ACM					CM				
	B	SE	χ ^2^	*p*-value	HR (95% CI)	B	SE	χ ^2^	*p*-value	HR (95% CI)
Age	0.028	0.006	22.156	<0.001	1.028 (1.016–1.040)	0.020	0.007	9.526	0.002	1.021 (1.007–1.034)
Sex	–0.074	0.149	0.250	0.617	0.928 (0.694–1.243)	–0.094	0.167	0.315	0.575	0.911 (0.657–1.263)
Smoking	0.006	0.153	0.002	0.967	1.006 (0.745–1.359)	–0.102	0.172	0.349	0.554	0.903 (0.645–1.266)
Drinking	–0.065	0.162	0.160	0.689	0.937 (0.681–1.288)	0.002	0.181	0.000	0.993	1.002 (0.703–1.427)
DM	0.066	0.141	0.219	0.640	1.068 (0.811–1.407)	0.158	0.155	1.039	0.308	1.171 (1.865–1.585)
Hypertension	0.166	0.123	1.819	0.177	1.181 (0.927–1.504)	0.111	0.138	0.647	0.421	1.118 (1.852–1.466)
TG	0.052	0.047	1.226	0.268	1.053 (0.961–1.154)	0.007	0.056	0.014	0.905	1.007 (0.902–1.123)
TC	0.067	0.095	0.486	0.486	1.069 (0.886–1.289)	0.150	0.104	2.097	0.148	1.162 (1.948–1.424)
HDL-C	0.018	0.114	0.024	0.876	1.018 (0.814–1.273)	–0.013	0.138	0.009	0.924	0.987 (0.752–1.294)
LDL-C	–0.151	0.112	1.822	0.177	0.859 (0.690–1.071)	–0.211	0.123	2.975	0.085	0.809 (0.637–1.029)
FGR high *vs.* low	0.448	0.121	13.846	<0.001	1.566 (1.236–1.983)	0.513	0.135	14.482	<0.001	1.670 (1.282–2.174)

*p* < 0.05 was statistically significant. ACM, all-cause mortality; CM, cardiogenic mortality; HR, hazard ratio; B, regression coefficient; SE, standard error; CHD, coronary heart disease; DM, diabetes mellitus; TG, triglycerides; TC, total cholesterol; HDL-C, high-density lipoprotein cholesterol; LDL-C, low-density lipoprotein cholesterol; FGR, fibrinogen/γ-glutamine transferase.

**Fig. 4.**
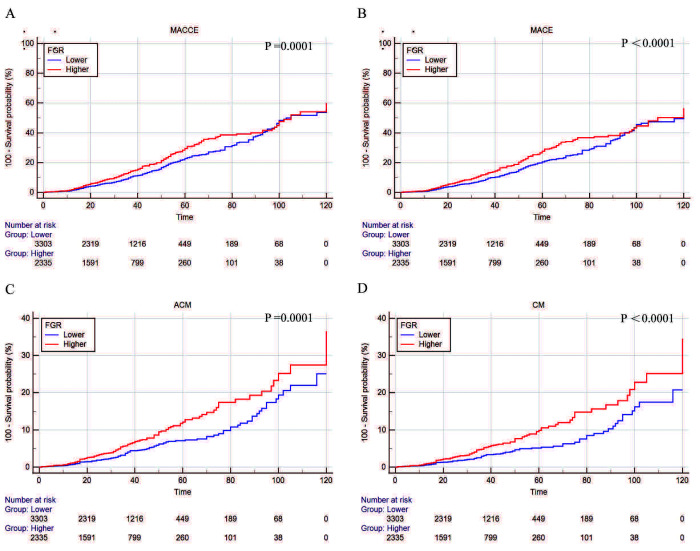
**Kaplan–Meier curves were plotted according to the follow-up endpoints**. (A) MACCEs. (B) MACEs. (C) ACM. (D) CM. MACCEs, major cardiac and cerebrovascular adverse events; MACEs, major adverse cardiovascular events; ACM, all-cause mortality; CM, cardiogenic mortality; FGR, fibrinogen/γ-glutamine transferase.

In the Kaplan–Meier curves shown in Fig. 4, the risk of events was significantly increased in the high FGR group. As the FGR ratio increased, the risk of adverse cardiac and cerebrovascular events increased, and the risk of all-cause and cardiac death was higher.

**Fig. 5.**
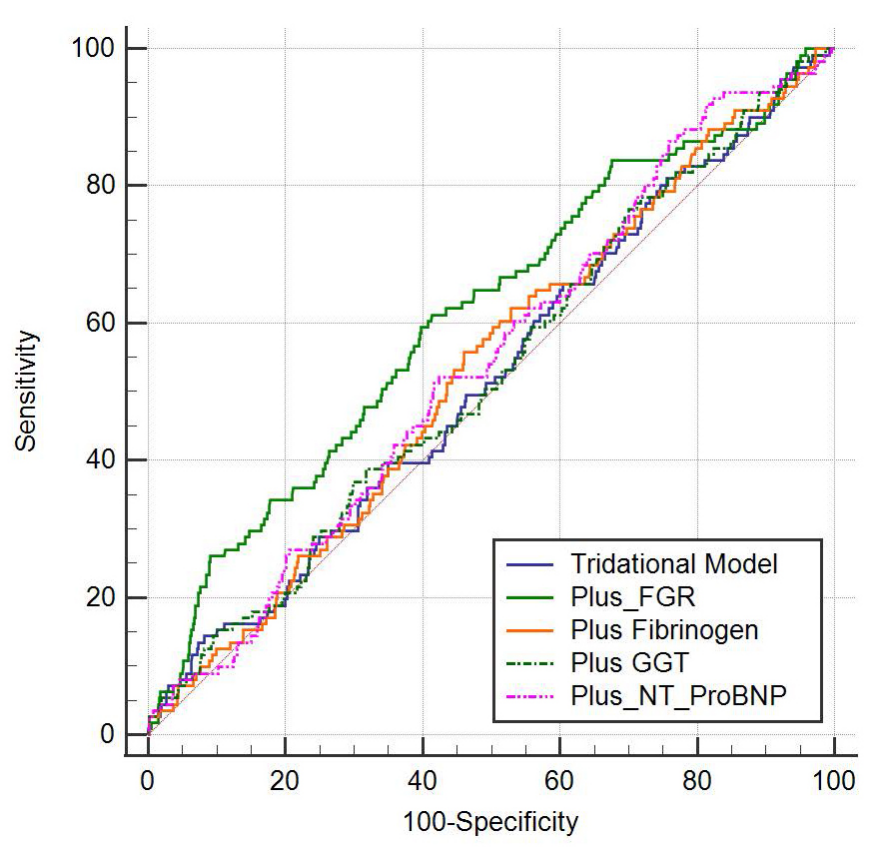
**Comparison of the risk of ACM in FGR and traditional risk factor models, Fibrinogen, GGT, and NT-proBNP**. The risk of ACM was statistically different between FGR and traditional risk factor models (*p* = 0.002), fibrinogen (*p* = 0.008), GGT (*p* = 0.004), and NT-proBNP (*p* = 0.024), the comparison between other different models was not statistically significant (*p* > 0.05). FGR, the fibrinogen to gamma-glutamine transferase ratio; GGT, gamma-glutamine transferase; NT-proBNP, N-terminal pro brain natriuretic peptide; ACM, all-cause mortality.

Traditional risk factors (age, sex, smoking, drinking, history of diabetes, history of hypertension, total cholesterol, triglycerides, HDL-C LDL-C) (**Supplementary Table 1A**) and fibrinogen (**Supplementary Table 1C**), GGT (**Supplementary Table 1D**), NT-proBNP (**Supplementary Table 1E**), and FGR indicators (**Supplementary Table 1B**) were included in the logistic regression model to observe the correlation between the above indicators and ACM events. The predictive performance of the occurrence of ACM events between FGR and traditional risk factors, fibrinogen, GGT, and NT-proBNP was compared by ROC curves. It was found that FGR was statistically different from the traditional risk factor model (*p* = 0.002), Fibrinogen (*p* = 0.008), GGT (*p* = 0.004), and NT-ProBNP (*p* = 0.024) in terms of risk of developing ACM; the comparison between other different models was not statistically significant (*p* > 0.05). The area under the FGR model curve was larger than that of the traditional risk factors, fibrinogen, GGT and NT-ProBNP models (Fig. 5).

## 4. Discussion

In this study, we found that high FGR was related to risk of long-term MACCE, long-term death and cardiogenic death in patients with CHD, with the use of a large sample database at 10 years of long-term follow-up. Thus, FGR can be used as a new predictor of long-term prognosis in patients with coronary heart disease. The prediction of the long-term prognosis of patients with coronary heart disease by simple indicators will benefit patients with CHD.

In a previous Framingham study of 1315 individuals with no prior cardiovascular disease who were followed for 12 years; 165 males and 147 females developed cardiovascular disease, and both the risk of cardiovascular disease and the risk of CHD were positively correlated with fibrinogen levels [7]. In Europe, according to a previous study of 3043 patients with angina pectoris coronary angiography examinations, after 2 years of follow-up and during the follow-up, 837 patients underwent coronary artery bypass grafting (CABG), 223 patients underwent coronary artery angioplasty, 49 individuals simultaneously received 2 types of treatment and there was a total of 106 cases of MACE. Furthermore, it was observed that there were more patients with myocardial infarction or sudden death with an increased fibrinogen concentration; this result was also more significant in patients with high cholesterol [8]. Ma J and other researchers investigated fibrinogen and future prospective risks of acute myocardial infarction [9]. They included 14,916 male patients aged 40–84 years-old; after 5 years of follow-up, 199 cases of acute myocardial infarction occurred, and they observed that a high baseline fibrinogen level was associated with a greater risk of acute myocardial infarction. Additionally, the high fibrinogen group had a two-fold added risk of acute myocardial infarction compared with the control group [9].

Other clinical studies have also found that fibrinogen levels are associated with coronary artery severity and stenosis in patients with CHD [10, 11, 12]. Fibrinogen can be used as an independent risk factor for coronary artery severity and poor prognosis in patients with CHD and can also be used as a novel biomarker. The increase or decrease in fibrinogen is a reflection of coagulation function and may lead to thrombosis and bleeding events. The mechanism of poor prognoses in patients with coronary heart disease caused by fibrinogen may be due to the following reasons: (1) It may promote the formation of thrombosis. Fibrinogen is decomposed into fibrin under the action of thrombin to participate in the coagulation process; additionally, it activates platelets to trigger the coagulation positive feedback process [13]. Fibrinogen can bind to the plasminogen receptor, thus rendering plasminogen-induced thrombolysis ineffective and leading to a decrease in clot solubility [14, 15]. Moreover, fibrinogen induces platelet aggregation and increases blood viscosity [16]. Thus, the combination of the above factors leads to thrombosis. (2) Fibrinogen promotes inflammatory factors. Fibrinogen promotes inflammatory responses by inducing pro-inflammatory factors (Tumor Necrosis Factor-α (TNF-α) and Interleukin-1β (IL-1 β)) on monocytes, and chemokines on endothelial cells and fibroblasts [17, 18, 19]. The accumulation of fibrinogen in the vascular wall promotes the infiltration of macrophages, which are the precursors of foam cells. Fibrinogen also activates platelets via glycoprotein IIb/IIIa receptors, thereby enhancing inflammatory responses. Additionally, activated platelets produce the proinflammatory cytokines IL-1β and CD40 ligands that are involved in the development and progression of atherosclerotic lesions [20]. (3) Fibrinogen promotes the formation of atherosclerosis. Fibrinogen increases the expression of intercellular adhesion molecule-1, thus leading to the increased adhesion of leukocytes, macrophages and platelets [21]. Moreover, fibrinogen deposition adsorbs LDL cholesterol and promotes atherosclerotic plaque formation [22].

The fibrinogen level can reflect the hypercoagulable state of blood and participate in the formation of thrombi. Furthermore, the fibrinogen level is also the main factor determining blood viscosity and red blood cell aberration and reflects the degree of atherosclerotic vascular injury [23, 24]. Fibrinogen may be involved in atherosclerotic diseases by affecting platelet aggregation [25] or by increasing blood viscosity, thus promoting thrombosis and leading to acute vascular thrombotic events, which promotes the development of atherosclerotic lesions [26, 27].

GGT was initially observed to be an indicator of hepatobiliary dysfunction and alcohol abuse. With subsequent epidemiological and pathological studies, GGT was also found to play a specific role in the pathogenesis and clinical prognosis of cardiovascular diseases caused by atherosclerosis. An Austrian epidemiological study of 163,944 Austrian adults who were followed for 17 years found that high GGT was significantly associated with an increase in overall mortality from cardiovascular disease [28]. Moreover, a systematic review of GGT and all-cause and cardiogenic death from CHD by Yang P *et al*. [29] showed that elevated serum GGT levels were an independent predictor of cardiogenic and all-cause mortality in CHD patients. Additionally, Long Y *et al*. [30] conducted a study of GGT and examined all-cause death and cardiovascular disease death. The study included 35 studies, and 571,511 patients were analyzed. The results showed that a total of 72,196 patients died and that GGT was associated with increased all-cause mortality and cardiovascular mortality. Moreover, there was a relationship between patients with coronary heart disease and type 2 diabetes, and all-cause mortality and cardiovascular mortality were associated with GGT. Furthermore, a study by Ndrepepa G *et al*. [31] on GGT activity and prognoses in patients with coronary heart disease showed that increased GGT activity was associated with an increased risk of 3-year all-cause, cardiac and non-cardiac death in 5501 patients with coronary heart disease after 3 years of follow-up. A study by Zheng YY *et al*. [32] on GGT and heart failure in patients with coronary heart disease after percutaneous coronary intervention (PCI) showed that serum GGT concentration was independently associated with heart failure after PCI; additionally, baseline GGT levels lower than 19.6 or ≥32.9 were found to increase the risk of heart failure in patients with coronary heart disease after PCI. Therefore, GGT not only serves as an independent predictor of death in patients with coronary heart disease, but also plays a role in the poor prognosis of patients with coronary heart disease or after PCI.

GGT may be associated with poor prognoses in CHD patients by the following mechanisms: (1) GGT was found in carotid atherosclerotic plaques after carotid endarterectomy [33], and a histochemical analysis showed that GGT was active and expressed in CD68+ macrophage-derived foam cells in human atherosclerotic plaques [34]. Additionally, it was found to co-localize with immunoactivity oxidize low-density lipoprotein (LDL) [35], and catalytically active GGT has been shown to adhere to microthrombi on atherosclerotic surfaces [36]. Moreover, GGT activity associated with lipoprotein (LDL, intermediate density lipoprotein (IDL) and very low-density lipoprotein (VLDL)) increases with total serum GGT activity, and increased serum GGT levels may be associated with the increased entry of GGT-carrying lipoproteins into plaques [37, 38]. (2) Oxidative stress is linked with GGT. During GGT activity, mercaptan metabolites produced on the cell surface can induce and promote oxidation reactions, thus leading to the generation of free radical oxidant species [39]. The production of cysteine-glycine in GSH catabolism mediated by GGT may promote the reduction of trivalent Fe to divalent Fe to form Fe pools and predispose to LDL oxidation [36]. (3) Inflammation is linked to GGT. GGT may produce leukotriene inflammatory factors through the 5-lipoxygenase pathway of arachidonic acid metabolism [40], or can be strongly correlated with serum C-reactive protein (CRP) concentrations [41].

Studies have shown that GGT may play a role in the cellular processes of LDL oxidation and atherosclerosis formation. Whitfield JB *et al*. [42] found that GGT may promote the formation and rupture of atherosclerotic plaques by catalyzing the oxidation of LDL. GGT plays an important role in atherosclerotic plaque formation and fibrous cap formation, the apoptosis of pathological cell components, plaque erosion and rupture, enhancement of platelet aggregation and thrombosis [43].

In this study, we combined two indicators, fibrinogen and GGT, and predicted the poor prognosis of CHD by their ratio. This study is a single-center, large-sample, retrospective cohort study. We retrospectively analyzed and followed up 5638 patients with CHD for a mean follow-up period of 35.9 ± 22.5 months, with the longest follow-up period being up to 10 years. We believe that FGR can be used as a myocardial marker for poor prognosis in patients with CHD for the following reasons: first, basic and clinical studies on fibrinogen and GGT suggest that they correlate with death, lesion extent, and poor prognosis in patients with CHD; however, in clinical practice, we found that there are many causes of elevated fibrinogen as well as GGT, which cannot be ruled out individually. Therefore, in this study, the ratio of fibrinogen to GGT was used to predict the poor prognosis of CHD, excluding the influence of some other adverse factors. FGR could be a more reliable prognostic indicator of CHD. Second, fibrinogen and GGT are easily available and widely used in clinical practice, and their predictive values are acceptable. FGR can be used as a complementary index for their clinical application to further enhance their reliability. Finally, according to the ROC curve in Fig. 5, the Area Under Curve (AUC) of FGR was larger than that of the individual indicators’ fibrinogen and GGT; similarly, the AUC of FGR was larger than that of conventional risk factors and NT-proBNP, and the *p* value was less than 0.05. Therefore, we conclude that FGR has a good predictive value. In conclusion, FGR can be used as a myocardial marker of poor prognosis in patients with CHD.

## 5. Conclusions

High FGR can increase the risk of MACCE, as well as long-term ACM and CM, in patients with CHD; additionally, it can be used as a new biomarker for long-term prognosis in patients with CHD.

## 6. Limitations

The limitation of this study is that the design type is a single-center retrospective cohort study. In the future, we will carry out a multi-center prospective study jointly with hospitals at other prefecture-level or other provinces and cities. Related cardiac markers, such as troponin, myoglobin, and cardiac enzymes, were not included in the study. Finally, we have only collected the results of hematological examinations during the hospitalization of the included subjects. If possible, we will contact the patients to redraw serum indicators to compare the changes of serological indicators before and after, so as to better identify the related risk factors.

## Data Availability

The datasets used and/or analyzed during the current study available from the corresponding author on reasonable request.

## Supplementary Material

Supplementary material associated with this article can be found, in the online version, at https://doi.org/10.31083/j.rcm2412369.
