# Supplementary material for: Predictive Value of the Fibrinogen to Gamma-Glutamine Transferase Ratio in the Long-Term Outcome in Patients with Coronary Heart Disease: A Retrospective Cohort Study
Source: Rev Cardiovasc Med. 2023 Dec 26;24(12):369. doi: 10.31083/j.rcm2412369 (PMC11272865; doi:10.31083/j.rcm2412369)
Supplement: Supplementary file 1 [file 2153-8174-24-12-369-s1.zip › 2153-8174-24-12-369-s1.docx]

**Supplementary Table 1A. Results of logistic regression analysis of traditional risk factors for ACM in patients with CHD.**

|  | ACM | | | | |
| --- | --- | --- | --- | --- | --- |
|  | B | SE | Wald | *p* | HR (95% CI) |
| Age | 0.034 | 0.006 | 31.605 | 0.000 | 1.03（1.02-1.05） |
| Sex | -0.097 | 0.152 | 0.450 | 0.525 | 0.91（0.67-1.22） |
| Smoking | 0.088 | 0.156 | 0.316 | 0.574 | 1.09（0.80-1.48） |
| Drinking | -0.064 | 0.164 | 0.152 | 0.696 | 0.94（0.68-1.29） |
| [DM](javascript:;) | -0.046 | 0.142 | 0.105 | 0.746 | 0.96（0.72-1.26） |
| Hypertension | 0.015 | 0.124 | 0.014 | 0.907 | 1.02（0.80-1.29） |
| TG | -0.004 | 0.050 | 0.006 | 0.941 | 1.00（0.90-1.10） |
| TC | 0.121 | 0.097 | 1.571 | 0.210 | 1.13（0.93-1.37） |
| HDL-C | 0.018 | 0.114 | 0.024 | 0.876 | 1.02（0.81-1.27） |
| LDL-C | -0.167 | 0.115 | 2.124 | 0.145 | 0.85（0.68-1.06） |
| Constant | -5.034 | 0.454 | 123.081 | 0.000 | 0.01（-） |

*p* < 0.05 was statistically significant.

**Supplementary Table 1B. Results of logistic regression analysis of traditional risk factors and FGR for ACM in patients with CHD.**

|  | ACM | | | | |
| --- | --- | --- | --- | --- | --- |
|  | B | SE | Wald | *p* | HR (95% CI) |
| Age | 0.033 | 0.006 | 29.934 | 0.000 | 1.03(1.02-1.05) |
| Sex | -0.102 | 0.153 | 0.441 | 0.506 | 0.90(0.67-1.22) |
| Smoking | 0.087 | 0.158 | 0.307 | 0.579 | 1.09(0.80-1.49) |
| Drinking | -0.054 | 0.165 | 0.108 | 0.742 | 0.95(0.69-1.31) |
| [DM](javascript:;) | -0.024 | 0.144 | 0.027 | 0.869 | 0.98(0.74-1.30) |
| Hypertension | -0.021 | 0.126 | 0.027 | 0.868 | 0.98(0.77-1.25) |
| TG | 0.027 | 0.050 | 0.281 | 0.596 | 1.03(0.93-1.13) |
| TC | 0.111 | 0.098 | 1.283 | 0.257 | 1.12(0.92-1.36) |
| HDL-C | 0.013 | 0.123 | 0.012 | 0.915 | 1.01(0.80-1.29) |
| LDL-C | -0.155 | 0.117 | 1.774 | 0.183 | 0.86(0.68-1.08) |
| FGR | 0.393 | 0.124 | 10.059 | 0.002 | 1.48(1.16-1.89) |
| Constant | -5.205 | 0.463 | 126.599 | 0.000 | 0.01(-) |

*p* < 0.05 was statistically significant.

**Supplementary Table 1C. Results of logistic regression analysis of traditional risk factors and fibrinogen for ACM in patients with CHD.**

|  | ACM | | | | |
| --- | --- | --- | --- | --- | --- |
|  | B | SE | Wald | *p* | HR (95% CI) |
| Age | 0.032 | 0.006 | 25.940 | 0.000 | 1.03（1.02-1.05） |
| Sex | -0.113 | 0.158 | 0.509 | 0.475 | 0.89（0.66-1.22） |
| Smoking | 0.056 | 0.162 | 0.121 | 0.728 | 1.060（0.77-1.46） |
| Drinking | 0.012 | 0.169 | 0.005 | 0.944 | 1.01（0.73-1.41） |
| [DM](javascript:;) | -0.071 | 0.148 | 0.228 | 0.633 | 0.93（0.70-1.25） |
| Hypertension | 0.070 | 0.129 | 0.296 | 0.587 | 1.07（0.83-1.38） |
| TG | 0.028 | 0.050 | 0.301 | 0.583 | 1.030（0.93-1.14） |
| TC | 0.070 | 0.102 | 0.468 | 0.494 | 1.07（0.88-1.31） |
| HDL-C | -0.011 | 0.131 | 0.007 | 0.935 | 0.99（0.77-1.28） |
| LDL-C | -0.151 | 0.121 | 1.571 | 0.210 | 0.86（0.68-1.09） |
| Fibrinogen | 0.180 | 0.059 | 9.253 | 0.002 | 1.20(1.07-1.35) |
| Constant | -5.453 | 0.521 | 109.459 | 0.000 | ＜0.01（-） |

*p* < 0.05 was statistically significant.

**Supplementary Table 1D. Results of logistic regression analysis of traditional risk factors and GGT for ACM in patients with CHD.**

|  | ACM | | | | |
| --- | --- | --- | --- | --- | --- |
|  | B | SE | Wald | *p* | HR (95% CI) |
| Age | 0.033 | 0.006 | 30.600 | 0.000 | 1.03(1.02-1.05) |
| Sex | -0.086 | 0.153 | 0.316 | 0.574 | 0.92(0.68-1.24) |
| Smoking | 0.095 | 0.158 | 0.364 | 0.546 | 1.10(0.81-1.50) |
| Drinking | -0.052 | 0.165 | 0.101 | 0.751 | 0.95(0.69-1.31) |
| [DM](javascript:;) | -0.022 | 0.144 | 0.023 | 0.878 | 0.98(0.74-1.30) |
| Hypertension | -0.010 | 0.126 | 0.006 | 0.939 | 0.99(0.77-1.27) |
| TG | 0.014 | 0.051 | 0.072 | 0.788 | 1.01(0.92-1.12) |
| TC | 0.123 | 0.098 | 1.602 | 0.206 | 1.13(0.94-1.37) |
| HDL-C | 0.014 | 0.122 | 0.014 | 0.906 | 1.01(0.80-1.29) |
| LDL-C | -0.169 | 0.116 | 2.155 | 0.146 | 0.85(0.67-1.06) |
| GGT | -0.004 | 0.003 | 2.031 | 0.154 | 1.00(0.99-1.00) |
| Constant | -4.925 | 0.463 | 113.113 | 0.000 | 0.01(-) |

*p* < 0.05 was statistically significant.

**Supplementary Table 1E. Results of logistic regression analysis of traditional risk factors and NT-proBNP for ACM in patients with CHD.**

|  | ACM | | | | |
| --- | --- | --- | --- | --- | --- |
|  | B | SE | Wald | *p* | HR (95% CI) |
| Age | 0.007 | 0.009 | 0.670 | 0.413 | 1.01(0.99-1.023) |
| Sex | 0.035 | 0.245 | 0.020 | 0.888 | 1.04(0.64-1.67) |
| Smoking | 0.197 | 0.243 | 0.658 | 0.417 | 1.22(0.76-1.96) |
| Drinking | 0.007 | 0.246 | 0.001 | 0.977 | 1.01(0.62-1.63) |
| [DM](javascript:;) | 0.268 | 0.211 | 1.614 | 0.204 | 1.31(0.87-1.98) |
| Hypertension | -0.038 | 0.196 | 0.037 | 0.847 | 0.96(0.66-1.41) |
| TG | 0.016 | 0.074 | 0.044 | 0.833 | 1.02(0.88-1.18) |
| TC | 0.052 | 0.145 | 0.129 | 0.720 | 1.05(0.79-1.40) |
| HDL-C | 0.097 | 0.150 | 0.416 | 0.519 | 1.10(0.82-1.48) |
| LDL-C | -0.126 | 0.173 | 0.533 | 0.465 | 0.88(0.63-1.24) |
| NT-proBNP | 0.000 | 0.000 | 11.544 | 0.001 | 1.00(1.00-1.00) |
| Constant | -3.571 | 0.679 | 27.702 | 0.000 | 0.03(-) |

*p* < 0.05 was statistically significant.
